# CD4 T‐cell hyporesponsiveness induced by schistosome larvae is not dependent upon eosinophils but may involve connective tissue mast cells

**DOI:** 10.1111/pim.12300

**Published:** 2016-01-28

**Authors:** C. T. Prendergast, D. E. Sanin, A. P. Mountford

**Affiliations:** ^1^ Centre for Immunology and InfectionDepartment of BiologyUniversity of YorkYorkUK; ^2^ Institute of InfectionImmunity and InflammationUniversity of GlasgowGlasgowUK; ^3^ Max‐Planck Institute of Immunobiology and EpigeneticsStübeweg51, 79108Freiburg im BreisgauGermany

**Keywords:** CD4 T lymphocytes < cell, Eosinophil < cell, Immune modulation < immunological terms, Mast cell < cell, Schistosomiasis < disease

## Abstract

In areas endemic for schistosomiasis, people can often be in contact with contaminated water resulting in repeated exposures to infective *Schistosoma mansoni* cercariae. Using a murine model, repeated infections result in IL‐10‐dependent CD4^+^ T‐cell hyporesponsiveness in the skin‐draining lymph nodes (sdLN), which could be caused by an abundance of eosinophils and connective tissue mast cells at the skin infection site. Here, we show that whilst the absence of eosinophils did not have a significant effect on cytokine production, MHC‐II^+^ cells were more numerous in the dermal cell exudate population. Nevertheless, the absence of dermal eosinophils did not lead to an increase in the responsiveness of CD4^+^ T cells in the sdLN, revealing that eosinophils in repeatedly exposed skin did not impact on the development of CD4^+^ T‐cell hyporesponsiveness. On the other hand, the absence of connective tissue mast cells led to a reduction in dermal IL‐10 and to an increase in the number of MHC‐II^+^ cells infiltrating the skin. There was also a small but significant alleviation of hyporesponsiveness in the sdLN, suggesting that mast cells may have a role in regulating immune responses after repeated exposures of the skin to *S. mansoni* cercariae.

**Abbreviations**
1x: single infection
4x: repeated infections
CFSE: carboxyfluorescein diacetate succinimidyl ester
DC: dendritic cells
DEC: dermal exudate cells
DTx: diphtheria toxin,
i.p.: Intraperitoneal
sdLN: skin‐draining lymph nodes
SSAP: soluble schistosomula antigen preparation

## Introduction

Schistosomiasis is a debilitating parasitic disease affecting approximately 230 million people worldwide caused by *Schistosoma* helminths 1, 2. Infection occurs after exposure of the skin to free‐swimming cercariae 3, and in areas that are endemic for this parasitic disease, individuals can be exposed to cercariae on numerous occasions during domestic activities, resulting in repeated infections. In this context, we developed a murine percutaneous infection model which showed that repeated exposure (4x) of the skin to infective *Schistosoma mansoni* cercariae resulted in hyporesponsiveness of CD3^+^ CD4^+^ T cells within the local skin‐draining lymph nodes (sdLN) 4. Significantly, this hyporesponsiveness was evident before the onset of egg deposition, which is conventionally associated with immune downregulation to chronic schistosome infection 5, 6, 7, 8, 9, 10, 11, and was dependent on the presence of IL‐10 without which CD4^+^ T cells in the sdLN were fully responsive to antigen 12.

After repeated *S. mansoni* infection, IL‐10 was predominantly produced by CD4^+^ T cells in both the sdLN 12 and the skin 13, yet the signals that trigger IL‐10 production by CD4^+^ T cells in this setting remain unclear. The skin infection site is the most likely cellular source of these IL‐10 inducing signals as it undergoes substantial changes after percutaneous exposure to infective cercariae including the influx of different immune cells (e.g. dendritic cells (DC), macrophages, eosinophils, neutrophils and CD3^+^ CD4^+^ T cells) 13, the proliferation of nonhaematopoietic cells (such as keratinocytes 4, 14) and major changes in the dermal cytokine environment 4, 14. One of the most noticeable effects in the skin of repeated schistosome infections is that up to 80% of dermal exudate cells (DEC) comprise SiglecF^+^ eosinophils 4. Eosinophils can have a significant effect on conditioning the immune response to many infectious diseases and in allergy 15, 16, and they have been considered important in the context of tissue remodelling and immune regulation 15, 17, 18, 19, 20. In general, eosinophils are thought to be host protective in defence against parasitic helminths; however, evidence can be contradictory, perhaps due to the numerous different methodologies available to investigate eosinophil function 21, 22, 23. Connective tissue mast cells, which differ from mucosal mast cells 24, are also present in significantly increased numbers in the skin after repeated schistosome infections 4. These cells are known to influence the regulation of the immune response by affecting antigen presentation, DC function and particularly T‐cell function 25. Therefore, we speculate that the abundance of eosinophils or mast cells could condition immune responses in the skin, and ultimately the development of CD4^+^ hyporesponsiveness in the lymph nodes draining the site of infection in mice exposed repeatedly to infective *S. mansoni* cercariae.

Here, we show that the abundant eosinophil population of DEC after repeated (4x) exposure to infective *S. mansoni* cercariae was significantly reduced following ablation using anti‐CCR3 mAb and was absent in eosinophil‐deficient ΔdblGATA‐1 mice. Somewhat surprisingly, however, despite eosinophils comprising the majority of 4x DEC, their absence did not have a major impact on the immune environment in the skin, or on the development of CD4^+^ T‐cell hyporesponsiveness in the sdLN. The role of connective tissue mast cells following repeated infection was investigated using mast cell‐deficient Mctp5Cre iDTR mice 26, 27, and we found that the absence of mast cells in the skin of 4x infected mice resulted in a reduction in the production of immunoregulatory IL‐10 by *in vitro* cultured skin biopsies, an increase in the number of MHC–II^+^ cells in the skin and led to a small but significant increase in the proliferation of cells recovered from the sdLN. This indicated that connective tissue mast cells could have a contributory role in the development of CD4^+^ T‐cell hyporesponsiveness within the sdLN.

## Materials and Methods

### Animals

Groups of C57BL/6 wild‐type (WT) mice and mast cell depletable Mcpt5cre iDTR mice 26 (obtained from Dr Axel Roers, Institute for Immunology, Technische Universität Dresden, Germany) were bred and housed at the University of York, York UK. ΔdblGATA‐1 mice (devoid of eosinophils) 28 were bred and housed at the University of Manchester, Manchester, UK. For both groups of transgenic mice, heterozygous littermates were used as infection controls. All experiments used age (6–10 weeks)‐ and sex‐matched animals carried out in accordance with the United Kingdom Animals Scientific Procedures Act 1986, and with approval of the University of York Ethics Committee, and/or the University of Manchester Ethics Committee.

### Parasites and infection protocol

Mice were percutaneously exposed via the pinnae to either a single dose (1x) of 75–150 *S. mansoni* cercariae 29, or to four doses (4x) 7 days apart 4. Given a 50% penetration rate to the pinna 4, the cumulative total infection dose in 4x mice was ~300 parasites per mouse.

### Eosinophil depletion by anti‐CCR3 antibody administration

Eosinophils were depleted from C57BL/6 mice with weekly doses of 1 mg rat anti‐CCR3 6S2‐19‐4 30 monoclonal antibody (mAb) in PBS via intraperitoneal (i.p.) injection. The ablation regime started 1 week prior to the first infection with *S. mansoni* cercariae, and the final dose taking place 24 h prior to the fourth infection. The anti‐CCR3 6S2‐19‐4 hybridoma cell line was a gift from Professor Judith Allen, Edinburgh University. The cell line was grown in a CELLine CL 1000 flask (Integra Biosciences, Zizers, Switzerland) and the mAb purified from the collected culture supernatant using an ÄKTA Prime.

### Mast cell depletion

Mcpt5Cre^+^ iDTR^+^ and Mcpt5Cre^−^ control mice received 25 ng/g diphtheria toxin (DTx, Sigma‐Aldrich, Dorset, UK) i.p. at weekly intervals for 4 weeks. Both groups of mice were then exposed to *S. mansoni* cercariae via the pinnae (as described above). During the infection time course, mice were also treated with 25 ng/g DTx i.p. at 7‐day intervals (1 day prior to infection), as well as DTx at the base of the ear subcutaneously (s.c.) at 5 ng/g (on the day of infection). Tissue sections of OCT embedded pinnae were stained with toluidine blue to determine the extent of mast cell depletion (Department of Veterinary Pathology, University of Liverpool).

### Recovery of dermal exudate cells (DEC)

Pinnae of mice were harvested 4 days after the final infection, split along central cartilage and floated on top of RPMI‐1640 (Gibco, Paisley, UK) containing 10% heat‐inactivated foetal calf serum (FCS) (Biosera, Uckfield, UK), 2 mm L‐glutamine, 1% Pen/Strep (both Gibco) and 50 μm 2‐mercaptoethanol (Sigma‐Aldrich) (complete RPMI) in nonadherent 24‐well tissue culture plates (Greiner Labortechnik, Frickenhausen, Germany) as previously described 4, 29. After an overnight culture *in vitro* at 37°C 5% CO_2_, DEC were recovered from the culture supernatant following centrifugation at 1000 g for 7 min at 4°C and resuspended in fresh complete RPMI. Cell‐free culture supernatants from the skin biopsies were stored at −20°C before analysis by cytokine‐specific ELISA.

### *In vitro* culture of total sdLN cells

Pairs of sdLN (auricular lymph nodes, draining the pinnae) were removed from mice 4 days after the final infection and were processed to a single‐cell suspension prior to culture at 1 × 10^6^ cells/mL in complete RPMI in the presence, or absence, of 50 μg/mL soluble schistosomula antigen preparation (SSAP) 31 for 72 h at 37°C. *In vitro* proliferation of sdLN cells was assessed through the decrease of carboxyfluorescein diacetate succinimidyl ester (CFSE) (Life Technologies, Paisley, UK) stain 4, 32. Briefly, sdLN cells were labelled with 3 μm CFSE prior to *in vitro* culture with parasite antigen. After 96 h of culture, sdLN cells were labelled with a fixable live/dead marker (Life Technologies) and then with fluorescently labelled anti‐CD3 and anti‐CD4 mAbs (eBioscience, Hatfield, UK); cell proliferation was measured as a decline in CFSE in the labelled cells determined by flow cytometry. Alternatively, proliferation was measured via incorporation of [^3^H]thymidine (18·5 kBq per well, PerkinElmer, Coventry, UK) by sdLN cells cultured *in vitro* with SSAP antigen as described previously 4, 32. Comparable results with regard to CD4^+^ T‐cell proliferation in the sdLN have been obtained previously with CFSE and [^3^H]thymidine incorporation 4, 12.

### Cytokine analysis by ELISA

Supernatants from skin biopsy cultures were collected for cytokine analysis as previously described 33. Cytokines IL‐4, IL‐10 and IL‐12p40 were quantified using the DuoSet ELISA kits (R&D Systems, Abingdon, UK).

### Flow cytometry

Cells were washed in ice‐cold PBS and labelled with LIVE/DEAD Fixable Aqua Dead Cell Stain (Life Technologies) according to the manufacturer's instructions. Cell aliquots were then washed in buffer (PBS containing 1% FCS), incubated with 1 μg anti‐CD16/32 mAb (eBioscience) in goat serum (Sigma‐Aldrich) and subsequently labelled with the following mAb conjugated to various fluorochromes: anti‐CD45 (clone 2D1), anti‐CD11b (clone M1/70), anti‐MHC‐II (IA‐IE) (clone M5/114), anti‐F4/80 (clone BM8), anti‐SiglecF (clone eBio440c), anti‐CD4 (clone RM4‐5) and anti‐CD3 (clone 17A2) (all eBioscience). Results were acquired on either a Cyan ADP analyser (DakoCytomation, Ely, UK), or a BD LSR Fortessa analyser (BD Biosciences, Oxford, UK). Data were analysed using flowjo software v7.6.5 (Tree Star Inc, Oregon Bio, Ashland, OR, USA).

### Statistics

Statistical analyses were performed using the Student's *t*‐test or one‐way analysis of variance (anova) test. Data on graphs are shown as mean ± SEM. Level of statistical significance is indicated in each figure. Statistical analyses and presentation of data were performed using graphpad prism 6 (GraphPad, Software Inc, San Diego, CA, USA).

## Results

**Figure 1:**
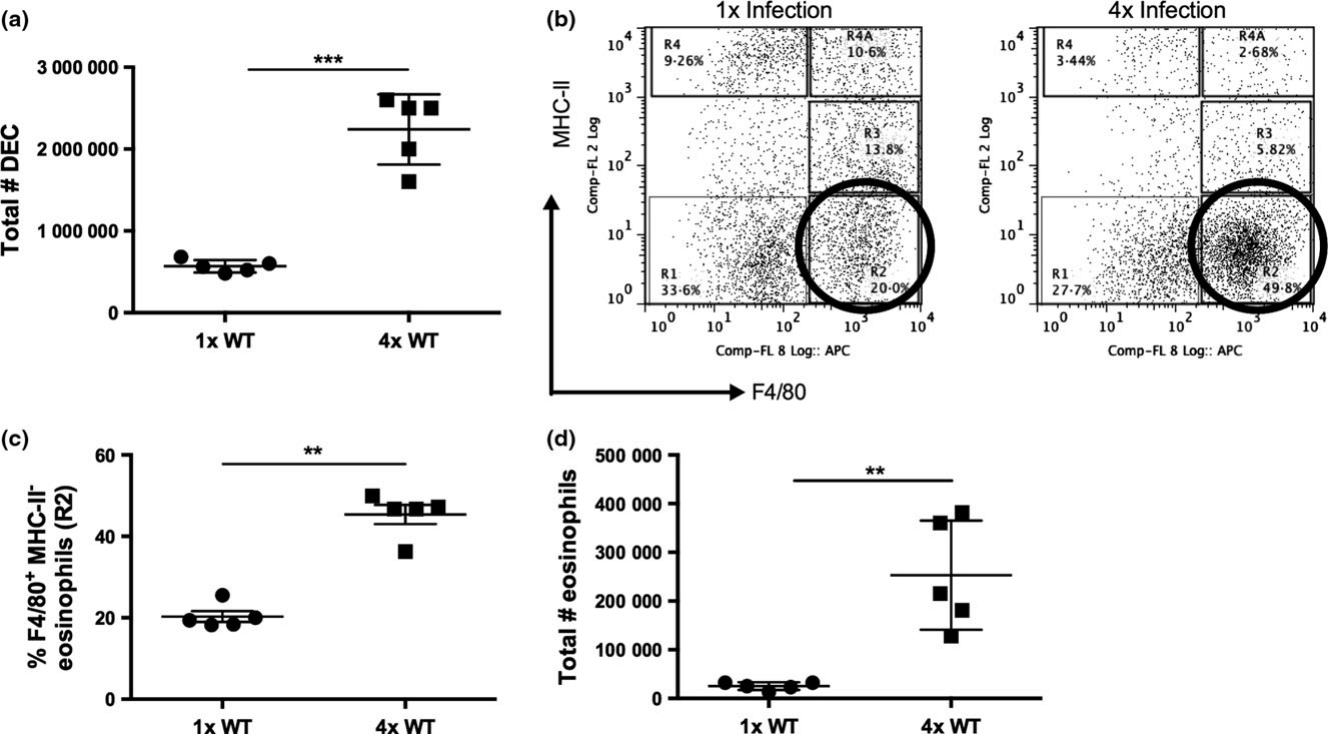
Eosinophils represent a significant proportion of the DEC population after repeated infections of the skin with *S. mansoni* cercariae. (a) Total number of DEC recovered from skin biopsies after 1x and 4x infection of C57BL/6 mice. (b) Representative flow cytometry dot plots of DEC previously gated on live cells, singlets and CD45^+^ cells showing expression of F4/80 and MHC‐II on DEC for 1x and 4x infected mice. Gating strategy is shown for ‘R1’ F4/80^−^MHC‐II^−^, ‘R2’ F4/80^+^MHC‐II^−^eosinophils, ‘R3’ F4/80^+^MHC‐II^int^ macrophages, ‘R4’ F4/80^−^MHC‐II^hi^ infiltrating DCs and ‘R4A’ F4/80^+^MHC‐II^hi^ tissue‐resident macrophages. Circles in bold highlight the ‘R2’ F4/80^+^ MHC‐II^−^ eosinophil population. (c) Proportion and (d) number of F4/80^+^ MHC‐II^−^ eosinophils in DEC after 1x and 4x infection. Symbols are values for individual mice within a single experiment, and horizontal bars are means ± SEM (*n* = 5 mice); ** denotes *P* ≤ 0·01, *** *P* ≤ 0·001 (Student's t‐test). Data are representative of four separate infection experiments.

Repeated infections (4x) of the skin with *S. mansoni* cercariae compared to a single infection (1x) resulted in an increased number of DEC recovered from *in vitro* cultured biopsies of the skin infection site (Fig. 1a). Based on the expression of F4/80 and MHC‐II, the recovered DEC population contained a mixture of cell types; the F4/80^−^ MHC‐II^−^ population (denoted ‘R1’) comprised cells which had previously been defined by our group as CD11b^+^ neutrophils and CD3^+^ CD4^+^ T cells 13, whilst the F4/80^+^ MHC‐II^−^ population (denoted ‘R2’) comprised eosinophils (Fig. 1b; ‘R2’ cell population is highlighted with circles in bold) previously shown to be SiglecF^+^ 4. The remaining cells were classed as macrophages or DCs based upon their differing expression levels of MHC‐II and F4/80 13, 34. Cells which were F4/80^+^ MHC‐II^int^ (denoted ‘R3’) were classified as macrophages, F4/80^−^ MHC‐II^hi^ cells (denoted ‘R4’) were classed as infiltrating DCs, whilst F4/80^+^ MHC‐II^hi^ cells (denoted ‘R4A’) were regarded as tissue‐resident macrophages (Fig. 1b). After 1x infection, the F4/80^+^ MHC‐II^−^ eosinophil population (R2) comprised approximately 20% of the CD45^+^ DEC population, whilst in 4x mice, there was a significant increase to ~40% of CD45^+^ DEC and eosinophils constituted the most abundant DEC population (Fig. 1b–d; *P* < 0·01).

**Figure 2:**
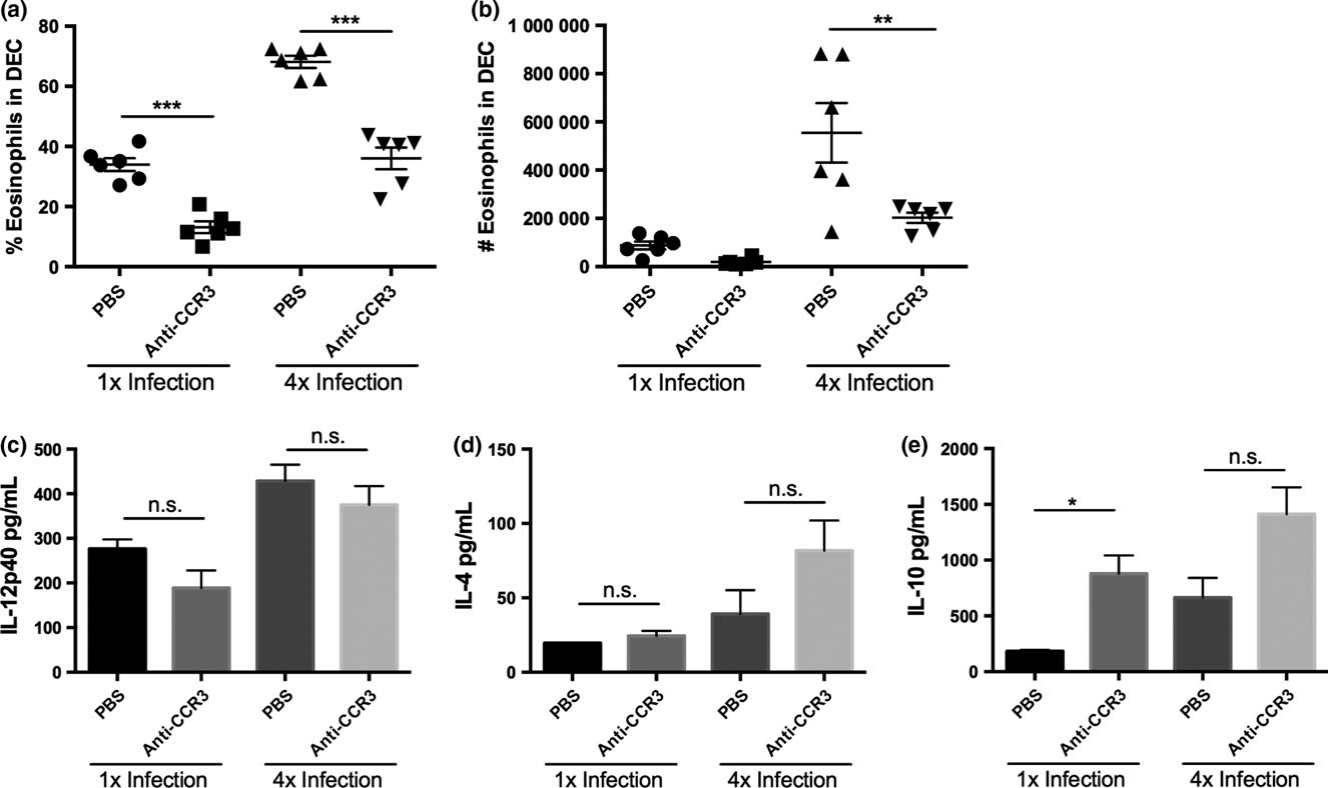
Administration of anti‐CCR3 mAb reduces eosinophilia in the skin. (a) Percentage, and (b) absolute number of eosinophils in DEC populations in 1x and 4x infected mice after anti‐CCR3 mAb treatment. Symbols are values for individual mice, and horizontal bars are means ±SEM (*n* = 6 mice); **P ≤ 0·01, *** *P* ≤ 0·001. Levels of (c) IL‐12p40, (d) IL‐4 and (e) IL‐10 released from *in vitro* cultured skin biopsies obtained from 1x and 4x infected mice. Bars represent mean + SEM; *P ≤ 0·05, n.s. = *P* > 0·05 (one‐way anova). Data are representative of three separate experiments.

To determine whether the abundance of eosinophils in the skin of 4x mice has an effect on the immune response at the site of infection and in the sdLN, anti‐CCR3 mAb was used to deplete eosinophils *in vivo*. Both the proportion (Fig. 2a) and number (Fig. 2b) of eosinophils in the DEC population were significantly reduced in 1x and 4x infected mice following anti‐CCR3 mAb treatment. However, cytokine production by *in vitro* cultured skin biopsies showed that the reduction in eosinophilia following anti‐CCR3 mAb treatment had no effect on the levels of IL‐12p40 and IL‐4 (Fig. 2c,d; *P* > 0·05), whilst there was a significant increase in IL‐10 only in 1x mice (Fig. 2e; 1x mice, *P* < 0·05; 4x mice, *P* > 0·05).

**Figure 3:**
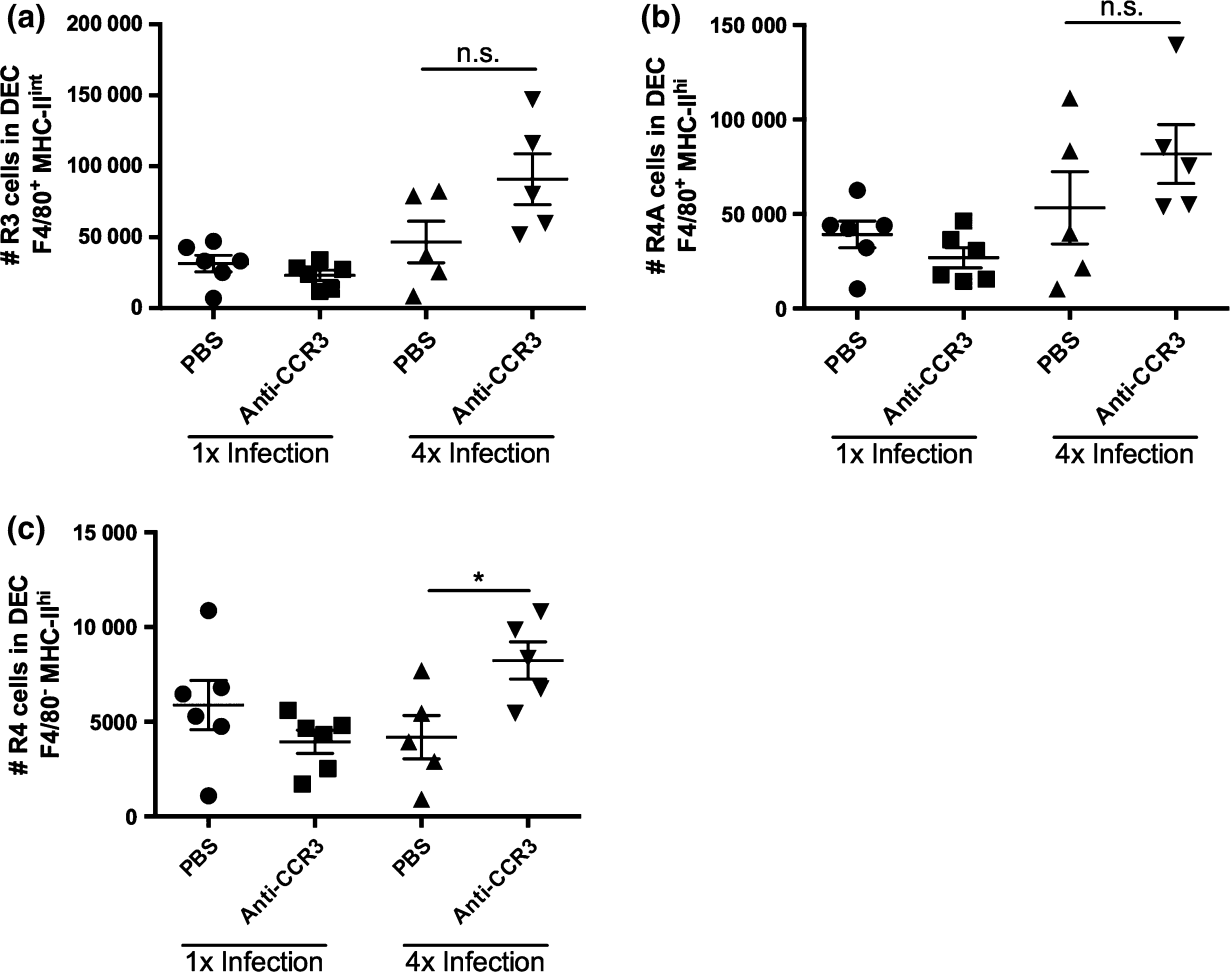
Depletion of eosinophils following administration of anti‐CCR3 mAb increases the number of infiltrating DCs. Numbers of (a) ‘R3’, F4/80^+^MHC‐II^int^ macrophages, (b) ‘R4A’ F4/80^+^MHC‐II^hi^ tissue‐resident macrophages and (c) ‘R4’ F4/80^−^MHC‐II^hi^ infiltrating DCs in DEC recovered from 1x or 4x mice after treatments with anti‐CCR3 mAb. Symbols are values for individual mice, and horizontal bars are means ± SEM (*n* = 5–6 mice); *P ≤ 0·05, where no value is given comparisons were not significant; *P* > 0·05 (one‐way anova). Data are representative of 3 separate experiments.

Eosinophil ablation by anti‐CCR3 mAb treatment revealed that there was a trend to increased numbers of both ‘R3’ F4/80^+^ MHC‐II^int^ macrophages, and ‘R4A’ F4/80^+^ MHC‐II^hi^ tissue‐resident macrophages in the DEC population obtained from 4x mice, although this was not significant (Fig. 3a,b; *P* > 0·05). There was, however, an increase in the number of ‘R4’ F4/80^−^ MHC‐II^hi^ infiltrating DCs in mAb‐treated 4x mice (Fig. 3c; *P* < 0·05).

**Figure 4:**
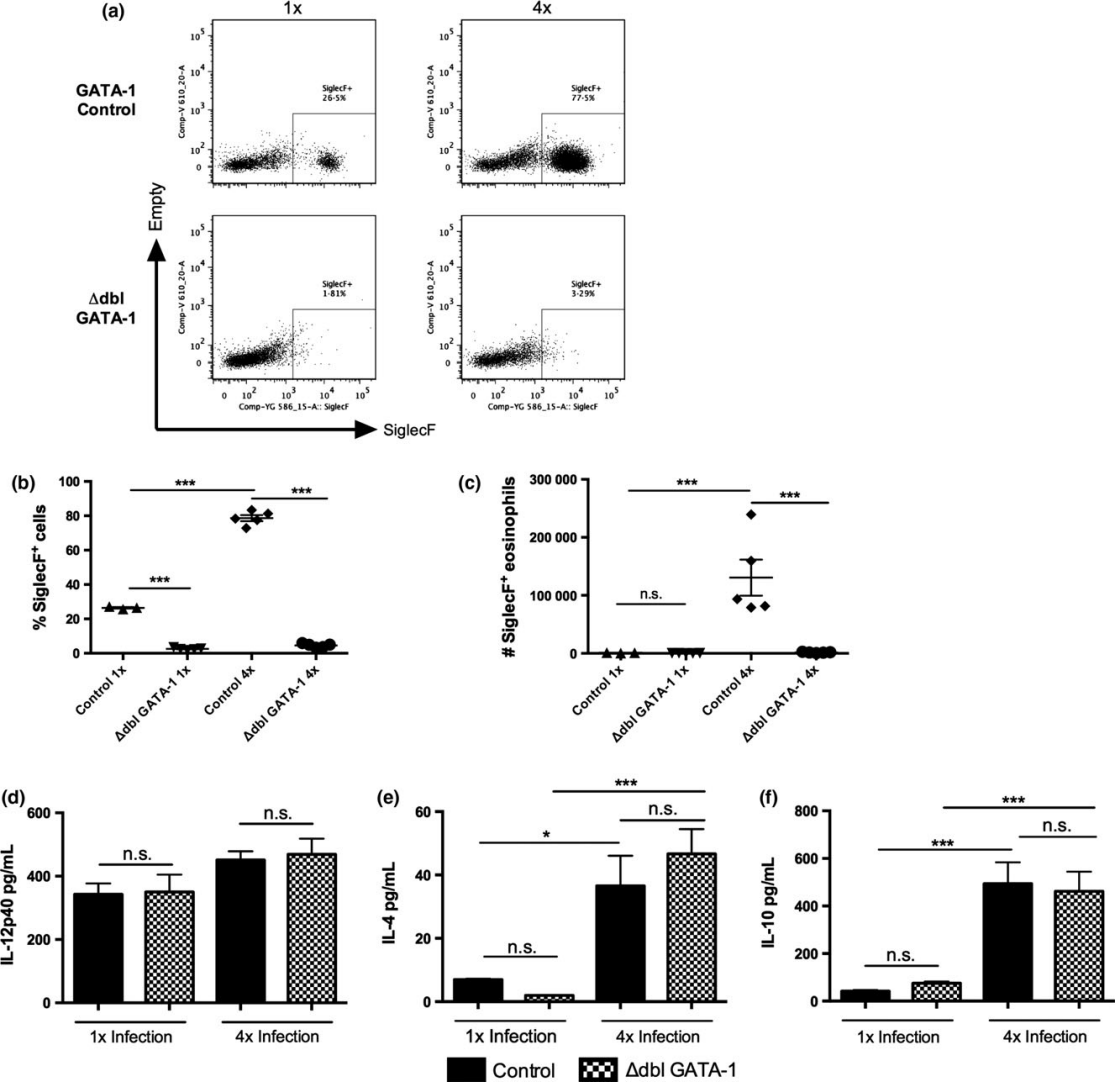
The absence of eosinophils in ΔdblGATA‐1 mice does not alter the production of IL‐12, IL‐4 or IL‐10 in the skin infection site. (a) Representative flow cytometry dot plots of DEC labelled with anti‐SiglecF^+^ to label eosinophil populations in 1x and 4x infected GATA‐1 control (top) and ΔdblGATA‐1 eosinophil‐deficient mice (bottom); gate denotes SiglecF^+^ cells. (b) Proportion and, (c) absolute number of SiglecF^+^ eosinophils in infected GATA‐1 control and ΔdblGATA‐1 eosinophil‐deficient mice. Symbols are values for individual mice; horizontal bars are means ± SEM (*n* = 3–5 mice). Production of (d) IL‐12p40, (e) IL‐4 and (f) IL‐10 by *in vitro* cultured skin biopsies from 1x and 4x infected mice. Error bars represent mean + SEM; *P ≤ 0·05, ***P ≤ 0·001, n.s. = *P* > 0·05 (one‐way anova).

As depletion of eosinophils in the skin after anti‐CCR3 mAb treatment was not absolute (Fig. 2a,b), immune responses were also examined in transgenic eosinophil‐deficient ΔdblGATA‐1 mice. Littermate GATA‐1‐sufficient control mice exhibited significant increases in the proportions and numbers of SiglecF^+^ eosinophils in 4x compared with 1x infected mice (Fig. 4a–c; *P* < 0·001). In contrast, eosinophils were absent in the ΔdblGATA‐1 mice after 1x and after 4x infections (Fig. 4a–c; *P* < 0·001). Analysis of cytokine production by *in vitro* cultured skin biopsies showed that whilst both IL‐4 and IL‐10 were significantly increased in 4x compared to 1x skin samples (Fig. 4e,f), there were no significant differences in the levels of IL‐4, IL‐10 or IL‐12p40, between those obtained from GATA‐1 control and ΔdblGATA‐1 mice (Fig. 4d–f).

**Figure 5:**
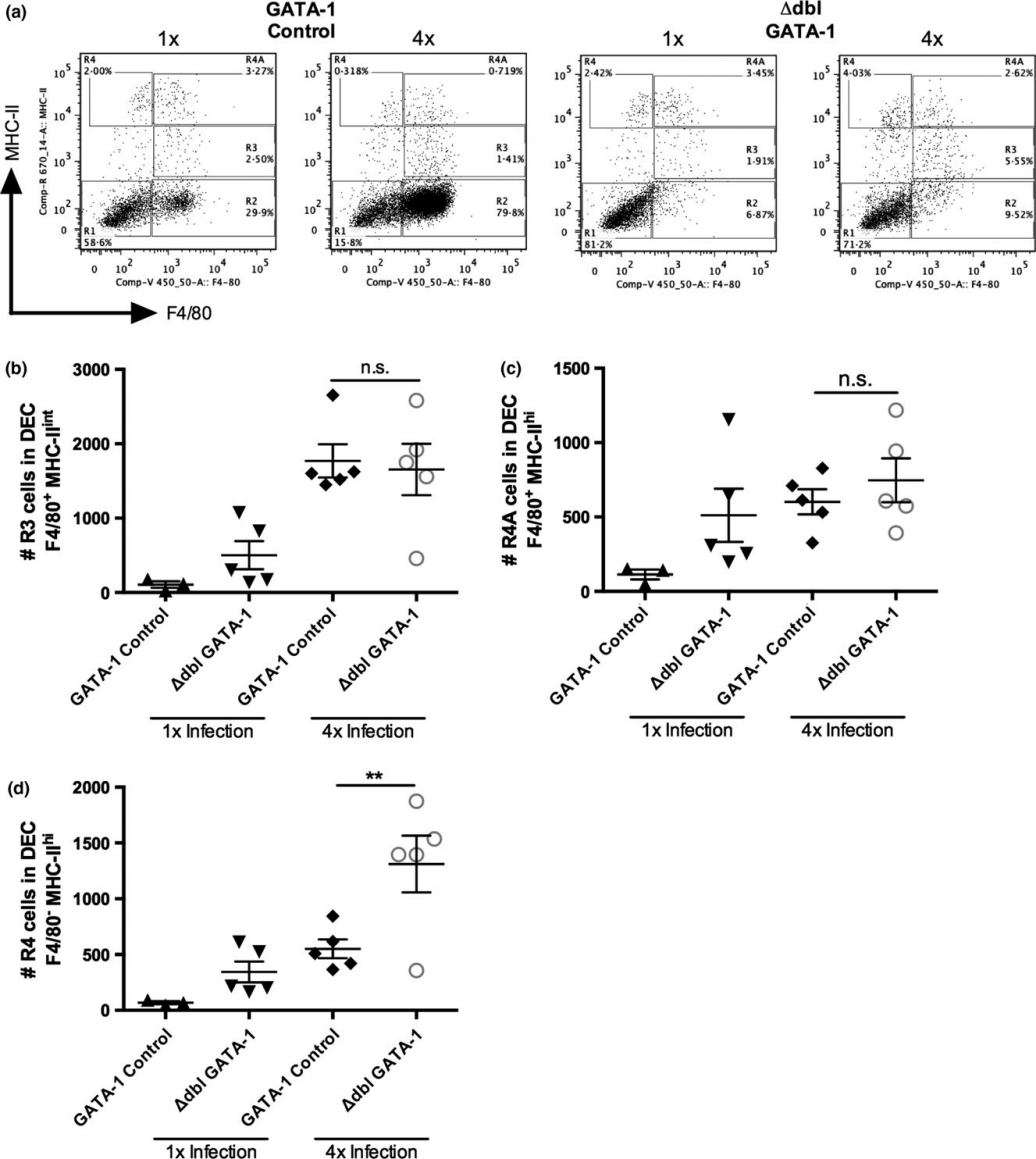
The absence of eosinophils in ΔdblGATA‐1 mice causes an increase in infiltrating DCs in the skin infection site of 4x mice. (a) Representative flow cytometry dot plots of cells previously gated on live cells and singlets, showing F4/80 and MHC‐II expression in DEC populations from 1x and 4x infected GATA‐1 control and ΔdblGATA‐1 eosinophil‐deficient mice. Numbers of (b) ‘R3’ (F4/80^+^MHC‐II^int^) macrophages, (c) ‘R4A’ (F4/80^+^MHC‐II^hi^) tissue‐resident macrophages and (d) ‘R4’ (F4/80^−^MHC‐II^hi^) infiltrating DCs in 1x and 4x infected mice. Symbols are values for individual mice, and horizontal bars are means ± SEM (*n* = 3–5 mice); **P ≤ 0·01, n.s. = *P* > 0·05 (one‐way anova).

The numbers of ‘R3’ (F4/80^+^ MHC‐II^int^) macrophages and R4A (F4/80^+^ MHC‐II^hi^) tissue‐resident macrophages recovered from 4x infected GATA‐1 control and ΔdblGATA‐1 mice were not significantly different (Fig. 5a–c; *P* > 0·05), although the numbers of R4 (F4/80^−^ MHC‐II^hi^) infiltrating DCs increased in 4x eosinophil‐deficient ΔdblGATA‐1 mice (Fig. 5d; *P* < 0·01). Therefore, the absence of the eosinophils appears to alter the number of infiltrating F4/80^−^ MHC‐II^hi^ DCs but not F4/80^+^ MHC‐II^+^ macrophages in the skin after repeated exposures to infective cercariae.

**Figure 6:**
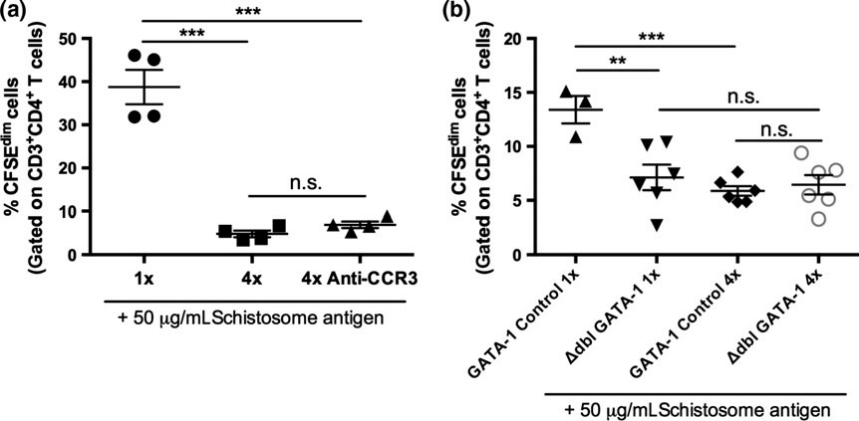
The absence of eosinophils does not alleviate CD4 T‐cell hyporesponsiveness in the sdLN of 4x infected mice. (a) Proportion of CFSE^dim^ CD3^+^ CD4^+^ T cells in 1x and 4x infected mice and 4x infected mice treated with anti‐CCR3 mAb, after *in vitro* stimulation with 50 μg/mL SSAP. CFSE^dim^ CD3^+^ CD4^+^ T cells are those that have undergone at least one round of proliferation. (b) Proportion of CFSE^dim^ CD3^+^ CD4^+^ T cells from 1x or 4x GATA‐1 control or ΔdblGATA‐1 eosinophil‐deficient mice, after *in vitro* culture with 50 μg/mL SSAP. Symbols are values for individual mice, and horizontal bars are means ± SEM (*n* = 3–6 mice); **P ≤ 0·01, ***P ≤ 0·001, n.s. = *P* > 0·05 (one‐way anova). Data are representative of 1–3 separate infection experiments.

The increase in the number of infiltrating MHC‐II^hi^ DC in the skin infection site in the absence of eosinophils (following anti‐CCR3 mAb treatment, and in eosinophil‐deficient ΔdblGATA‐1 mice) could enhance downstream antigen presentation to naïve T cells and therefore increase the responsiveness of CD4^+^ T cells in the sdLN. However, in response to *in vitro* re‐stimulation with parasite antigen, proliferation of CD4^+^ T cells from anti‐CCR3‐treated 4x infected mice was low and was comparable to proliferation from 4x infected PBS‐treated mice (Fig. 6a; *P* > 0·05). In fact, levels of CD4^+^ T‐cell proliferation in both groups of 4x infected mice were significantly lower than in 1x infected mice (Fig. 6a; *P* < 0·001). Low levels of CD4^+^ T‐cell proliferation were also observed in 4x eosinophil‐deficient ΔdblGATA‐1 mice, which were the same as the low levels in 4x GATA‐1 control mice (Fig. 6b) showing that the absence of eosinophils did not alleviate CD4^+^ T‐cell hyporesponsiveness.

**Figure 7:**
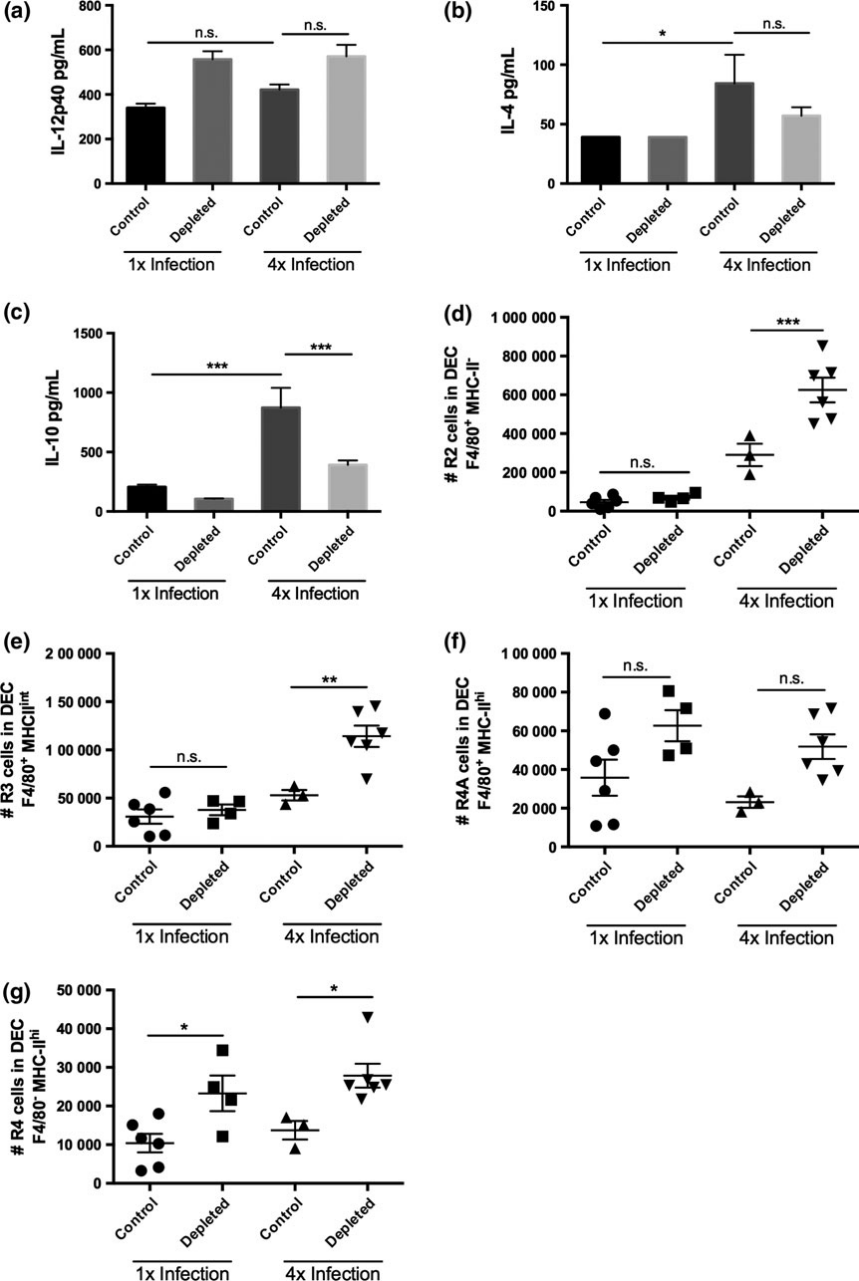
The absence of mast cells in the skin alters production of IL‐10 and increases the number of MHC‐II^+^ cells in the skin. Production of (a) IL‐12p40, (b) IL‐4 and (c) IL‐10, by *in vitro* cultured skin biopsies obtained from 1x and 4x infected Mcpt5Cre control and mast cell‐depleted mice. Numbers of (d) ‘R2’ (F4/80^+^MHC‐II^−^) eosinophils, (e) ‘R3’ (F4/80^+^MHC‐II^int^) macrophages, (f), ‘R4A’ (F4/80^+^MHC‐II^hi^) tissue‐resident macrophages and (g) ‘R4’ (F4/80^−^MHC‐II^hi^) infiltrating DCs in 1x and 4x infected mice. Symbols are values for individual mice, and horizontal bars are means ± SEM (*n* = 3–6 mice); *P ≤ 0·05, **P ≤ 0·01, ***P ≤ 0·001, n.s. = *P* > 0·05 (one‐way anova).

A possible role of connective tissue mast cells in conditioning the dermal immune environment was investigated in Mcpt5‐Cre^+^ iDTR^+^ mice 27, 35 depleted of mast cells from the skin through the administration of successive doses of diphtheria toxin (DTx). Near total ablation of mast cells as assessed by staining of skin sections with toluidine blue was achieved with four doses of DTx, (Fig. S1a,b), although a note of caution is required as degranulated mast cells would likely not be identified using this technique and could act as a potential source of immunological mediators. In the absence of mast cells, the production of IL‐12p40 and IL‐4 from 4x skin biopsies was unaffected compared to Mcpt5‐Cre^−^ control mice (Fig. 7a,b; both *P* > 0·05), although the production of regulatory IL‐10 by 4x infected Mcpt5‐Cre^+^ iDTR^+^ mice was significantly reduced, suggesting mast cells affect secretion of this cytokine (Fig. 7c, *P* < 0·001). Analysis of the DEC populations recovered from the skin infection site of 4x infected mice showed that there was an increase in the number of ‘R2’ F4/80^+^ MHC‐II^−^ eosinophils in Mcpt5‐Cre^+^ compared to Mcpt5‐Cre^−^ control mice (Fig. 7d, *P* < 0·001). There were also increases in the numbers of ‘R3’ F4/80^+^ MHC‐II^int^ macrophages (Fig. 7e; *P* < 0·01) and ‘R4’ F4/80^−^ MHC‐II^hi^ infiltrating DCs (Fig. 7g; *P* < 0·05), although the apparent increase in F4/80^+^ MHC‐II^hi^ tissue‐resident macrophages was not significant (Fig. 7f; *P* > 0·05).

**Figure 8:**
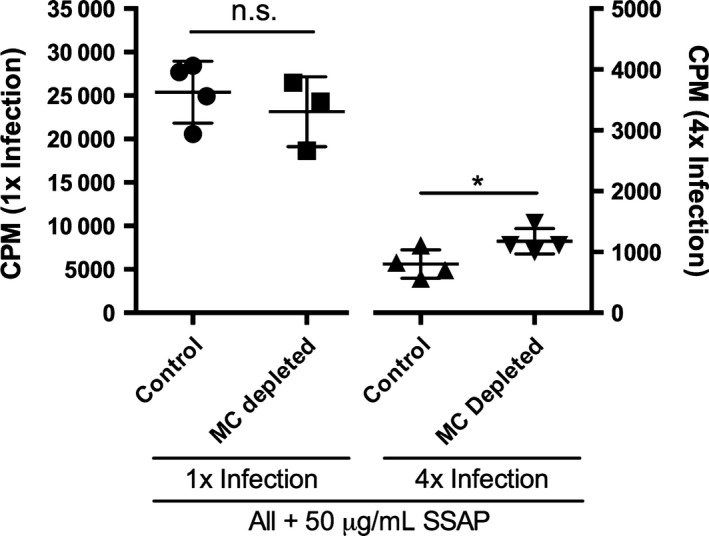
The absence of connective tissue mast cells in the skin leads to a slight increase in sdLN cell responsiveness. Proliferation of *in vitro* cultured sdLN cells stimulated with SSAP in 1x and 4x infected Mcpt5Cre^+^iDTR^+^ mast cell‐depleted and Mcpt5Cre^−^ control mice, as measured by [3H]thymidine incorporation. Error bars represent mean ± SEM; *n* = 4 mice per group, *P ≤ 0·05 (one‐way anova). Data are representative of two separate infection experiments.

Finally, there was no significant difference in the levels of *in vitro* SSAP‐specific proliferation of sdLN cells recovered from 1x infected mast cell‐depleted Mcpt5‐Cre^+^ compared to 1x infected control Mcpt5‐Cre^−^ mice (Fig. 8; *P* > 0·05). In contrast, the absence of mast cells in 4x infected mice resulted in a small but significant increase in cell proliferation compared to 4x infected control mice (Fig. 8; *P* < 0·05). This suggests that mast cells may have a limited role in regulating the inflammatory immune response as their absence results in a partial alleviation of the T‐cell hyporesponsiveness observed in the sdLN.

## Discussion

This study aimed to investigate the role of eosinophils and connective tissue mast cells in the early dermal immune response in a murine model of repeated exposures to infective *S. mansoni* cercariae. Both types of cells are abundant after multiple infections, and their presence may be critical to conditioning the local immune response, particularly the development of CD4 T‐cell hyporesponsiveness, which is a hallmark of repeated exposure to schistosome cercariae 4. The data presented here show that eosinophils do not have a major effect in conditioning the immune response after 4x infection. On the other hand, the absence of connective tissue mast cells was showed to alleviate CD4 T‐cell hyporesponsiveness, possibly by causing reduced production of IL‐10 in the skin.

Eosinophils are known to be elevated in allergy and in response to helminth infection, although their precise role is still being established 17, 36. Indeed, the absence of eosinophils adversely affects the survival of *Trichinella spiralis* helminth larvae, suggesting that they can be protective towards parasite infection 37. Previously we reported that the large influx of eosinophils into the skin infection site could be blocked by injection with rIL‐12, which also restored responsiveness of CD4^+^ T cells in the sdLN 4. We therefore hypothesized that eosinophils may indirectly condition the local immune response in the skin leading to CD4^+^ T‐cell hyporesponsiveness and examined this through direct ablation following administration of anti‐CCR3 mAb and in ΔdblGATA‐1 mice. We showed that there was no difference in the production of IL‐4, IL‐12p40 and IL‐10 in the skin of 4x infected mice using either strategy, although anti‐CCR3 mAb treatment did not yield complete eosinophil ablation leaving a small number of remaining cells which could be sufficient to negate any differences in cytokine production. This supports previous studies that found no difference in serum IL‐4 production between *S. mansoni*‐infected WT and ΔdblGATA‐1 mice 22.

Eosinophils can produce IL‐10 15, which is critical for T‐cell hyporesponsiveness in our model 12, and it has been reported that IL‐10 derived from eosinophils facilitates survival of the helminth parasite *T. spiralis* 38. So, it is perhaps surprising that we did not detect a significant reduction in IL‐10 cytokine production in the absence of eosinophils. However, we recently showed that in 4x schistosome‐infected mice, the majority of IL‐10^+^ cells in the skin were of lymphoid, rather than myeloid, origin 13. Thus, eosinophils are not a source of regulatory IL‐10 in our model despite their abundance.

The absence of eosinophils resulted in a significant increase in the numbers of infiltrating MHC‐II^hi^ DCs. This is counter to the view of others that the absence of eosinophils decreases the recruitment of DCs 38, 39, as eosinophil‐derived neurotoxin (EDN) acts as a DC chemoattractant *in vitro* and *in vivo* 40. The increase in the number of MHC‐II^hi^ DCs and their subsequent migration to the sdLN might be expected to result in enhanced CD4^+^ cell proliferation. However, the absence of eosinophils did not increase CD4^+^ cell responsiveness in the sdLN of 4x mice. On the other hand, as it is known that eosinophils can act directly as antigen‐presenting cells after infection with the helminth *Strongyloides* 41, 42, 43, it is possible that eosinophils aid priming CD4^+^ cell responses. Indeed, in 1x schistosome‐infected ΔdblGATA‐1 mice, the CD4^+^ T‐cell response in the sdLN was lower than in GATA‐1 control mice, indicating that after a single exposure to cercariae eosinophils might be acting by an undefined mechanism (e.g. antigen presentation, or cytokine production) to promote the initial immune response. Nevertheless, this reduction was not observed after anti‐CCR3 mAb was administered to 1x infected mice. Moreover, CD4^+^ T cells in the sdLN remained hyporesponsive in 4x infected ΔdblGATA‐1 mice. This supports earlier studies where anti‐IL‐5 mAb used to ablate eosinophils at the chronic stage of *S. mansoni* infection had no effect on the cellular immune response 44. Schistosome infection of eosinophil‐deficient ΔdblGATA‐1 and TgPHIL mice also had no effect on the disease process 22, although another study observed that infected ΔdblGATA‐1 mice had reduced Th2 response to *S. mansoni* eggs in terms of IL‐4 production 45.

As mast cells have the potential to impact on the development of acquired immune responses 24, 25, it was thought possible that connective tissue mast cells play a role in the development of CD4^+^ T‐cell hyporesponsiveness in our model, although in a recent report mast cells had no impact on the development of skin localized immune responses to *Leishmania major* 46. Here, we found that using schistosome‐infected Mcpt5Cre iDTR mice, there was an increase in the number MHC‐II^+^ cells in the absence of mast cells, supporting the notion that mast cells regulate cell populations with antigen‐presenting function 47. This could be related to the decrease in the amount of IL‐10 released in the skin of 4x Mcpt5‐Cre^+^ iDTR^+^ mast cell‐deficient mice leading to enhanced presentation of parasite antigen to CD4^+^ T cells in the sdLN. Therefore, as CD4^+^ hyporesponsiveness in the sdLN of 4x mice in our infection model is IL‐10 dependent 12, and the partial recovery in CD4^+^ T‐cell responsiveness in the 4x infected Mcpt5Cre iDTR mice was accompanied by a loss in the production of IL‐10, we propose mast cells contribute signals to condition dermal CD4^+^ T cells in the skin to produce further IL‐10.

In conclusion, whilst significantly increased numbers of eosinophils were observed in the skin after repeated exposure to *S. mansoni* cercariae, the absence of eosinophils had no bearing on CD4^+^ T‐cell responses in the downstream sdLN. Conversely, the absence of connective tissue mast cells led to decreased IL‐10 production and increased numbers of MHC‐II^+^ cells in the skin which promotes responsiveness of cells in the sdLN. Therefore, we suggest that connective tissue mast cells condition the skin cytokine environment, which dampens and downregulates CD4^+^ T‐cell responses in the sdLN leading to hyporesponsiveness.

## Author Contribution

CTP and DES performed the experiments and analysed the results, and CTP, DES and APM wrote the manuscript.

## Conflict of Interest

The authors declare no commercial or financial conflict of interest.

## Supporting information


**Figure S1.** Depletion of mast cells using Mcpt5Cre^+^iDTR^+^ mice requires the administration of four doses of DTx. (a) Representative images of Toluidine Blue stained sections of naïve ear pinnae to visualize mast cells after no DTx treatment (left), 1x DTx (middle) or 4x weekly DTx (right) treatment. Arrows represent mast cells. (b) Quantitative analysis of mast cell depletion following administration of DTx in either control (Mcpt5Cre^−^iDTR), or mast cell depletable (Mcpt5Cre^+^iDTR^+^) mice as determined by the number of mast cells in the field of view (F.O.V.) per pinnae section. Scale bar represents 0·1 mm. Symbols are values for individual mice, horizontal bars are means ± SEM (*n* = 4–7 mice); ***P ≤ 0·001, n.s. = *P* > 0·05 (one‐way anova).
