# Supplementary figures and images for: CD4 T‐cell hyporesponsiveness induced by schistosome larvae is not dependent upon eosinophils but may involve connective tissue mast cells
Source: Parasite Immunol. 2016 Jan 28;38(2):81–92. doi: 10.1111/pim.12300 (PMC4744672; doi:10.1111/pim.12300)

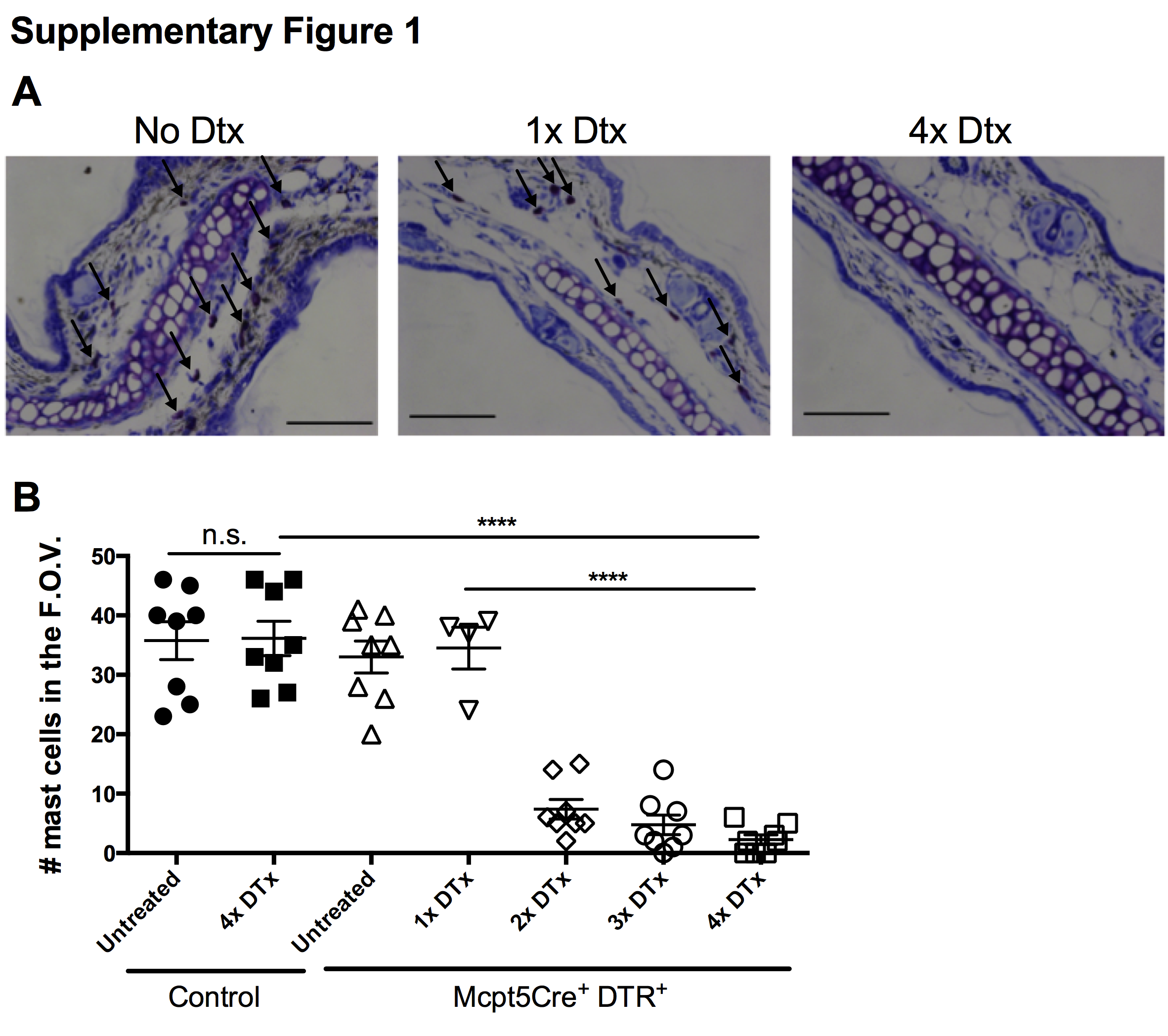

Supplement: Supplementary file 1 — Figure S1. Depletion of mast cells using Mcpt5Cre+iDTR+ mice requires the administration of four doses of DTx. (a) Representative images of Toluidine Blue stained sections of naïve ear pinnae to visualize mast cells after no DTx treatment (left), 1x DTx (middle) or 4x weekly DTx (right) treatment. Arrows represent mast cells. (b) Quantitative analysis of mast cell depletion following administration of DTx in either control (Mcpt5Cre−iDTR), or mast cell depletable (Mcpt5Cre+iDTR+) mice as determined by the number of mast cells in the field of view (F.O.V.) per pinnae section. Scale bar represents 0·1 mm. Symbols are values for individual mice, horizontal bars are means ± SEM (n = 4–7 mice); ***P ≤ 0·001, n.s. = P > 0·05 (one‐way anova). [file PIM-38-81-s001.tiff]
